# A comparison between the low back pain scales for patients with lumbar disc herniation: validity, reliability, and responsiveness

**DOI:** 10.1186/s12955-020-01403-2

**Published:** 2020-06-10

**Authors:** Min Yao, Bao-ping Xu, Zhen-jun Li, Sen Zhu, Zi-rui Tian, De-hua Li, Jue Cen, Shao-dan Cheng, Yong-jun Wang, Yan-ming Guo, Xue-jun Cui

**Affiliations:** 1 Spine Disease Institute, Longhua Hospital, Shanghai University of Traditional Chinese Medicine, 725 South Wanping Road, Shanghai, 200032 China; 2 Key Laboratory of Theory and Therapy of Muscles and Bones, Ministry of Education, Shanghai University of Traditional Chinese Medicine, 725 South Wanping Road, Shanghai, 200032 China; 3 Lu’an Hospital of Traditional Chinese Medicine, 76 Renmin Road, Anhui Lu’an, 237000 China; 4 Gansu Provincial Hospital of Traditional Chinese Medicine, 418 Guazhou Road, Lanzhou, 730050 Gansu China; 5 Department of Orthopaedic, Shanghai Pudong Gongli Hospital, Second Military Medical University, 219 Miaopu Road, Shanghai, 200013 China; 6 Shanghai Guanghua Hospital, 540 Xinhua Road, Shanghai, 200052 China

**Keywords:** Responsiveness, Minimal detectable change, Minimal clinically important difference, Low Back pain, Lumbar disc herniation

## Abstract

**Background:**

Although the Japanese Orthopedic Association Back Pain Evaluation Questionnaire (JOABPEQ), Numerical Pain Rating Scale (NPRS), Oswestry Disability Index (ODI), Roland Morris Disability Questionnaire (RMDQ), and Short Form 36 Health Survey (SF-36) has shown a preferable psychometric properties in patients with low back pain (LBP), but no study has yet determined these in conservative treatment of patients with lumbar disc herniation (LDH). Thus the current study aimed to compare those scales in LDH patients receiving conservative treatment to select the better option to assess the severity of disease.

**Methods:**

LDH patients were invited to complete the JOABPEQ, NPRS, ODI, RMDQ, and SF-36 twice. The internal consistency was evaluated by the Cronbach’s α. Test-retest reliability was tested by the intraclass correlation coefficient (ICC). The relationships of these scales were evaluated by the Pearson correlation coefficients (*r*). The responsiveness was operationalised using the receiver operating characteristic (ROC) curve, as well as the comparison of smallest detectable change (SDC), minimum important change (MIC).

**Results:**

A total of 353 LDH patients were enrolled. Four subscales of the Chinese JOABPEQ were over 0.70, then the ICCs for the test-retest reliability were over 0.75. For functional status, remarked negative correlations could be seen between JOABPEQ Q2-Q4 and ODI, as well as RMDQ (*r* = − 0.634 to − 0.752). For general health status, remarkable positive correlations could also be seen between Q5 Mental health and SF-36 PCS (*r* = 0.724) as well as SF-36 MCS (*r* = 0.736). Besides, the area under of the curves (AUC) of the JOABPEQ ranged from 0.743 to 0.827, indicating acceptale responsiveness, as well as the NPRS, ODI, and RMDQ.

**Conclusion:**

NPRS, and ODI or RMDQ is recommended in studies related to LDH patients, while if the quality of life also is needed to observe, the NPRS, and JOABPEQ would be more appropriate rather than SF-36.

## Introduction

Lumbar disc herniation (LDH) is one of the common causes of low back pain (LBP) [1, 2]. Symptomatic herniations present as lumbar radiculopathy including radicular pain, sensory abnormalities from both a mechanical compression and chemical irritation of the nerve root [2]. LDH occurs in approximately 10% of the population and has a serious impact on the work and life quality of patients and is the most common causes working-age individuals to undergo lumbar spine surgery, and also generates a large economic burden [3, 4].

Nevertheless, no objective biological markers are available to evaluate LDH severity, it is well known that the patient’s opinion of the results by patient-reported outcomes tools are still a very important measurement of treatment quality, several patient-reported outcomes tools were used to assess LBP such as the Numerical Pain Rating Scale (NPRS), and the Visual Analogue Scale (VAS) for pain intensity, the Roland Morris Disability Questionnaire (RMDQ), and the Oswestry Disability Index (ODI) for functional status, and the Short Form 36 Health Survey (SF-36) for general health status [5]. While the Japanese Orthopedic Association Back Pain Evaluation Questionnaire (JOABPEQ), it included five subscales including Q1 Low back pain for pain intensity, Q2 Lumbar function, Q3 Walking ability, Q4 Social life function for functional status, and Q5 Mental health for general health status, which is more comprehensive to assess pain intensity, functional status, and quality of life [6]. It was concluded that there were small correlations between JOABPEQ and NPRS, medium correlations between Q2 Lumbar function, Q3 Walking ability, Q4 Social life function and ODI, RMDQ, Short Form 8 Health Survey physical component summary (SF-8 PCS); and between Q5 Mental health and SF-8, SF-36, and EuroQol-5D (EQ-5D) in LBP patients or patients after lumbar surgery [7–10].

Although all of these scales has shown a preferable psychometric properties in patients with LBP, but no study has yet determined these psychometric properties in conservative treatment of patients with LDH [7, 10, 11]. The RMDQ is comprised of 24 items, the ODI is made up of 10 items, and the SF-36 consists of 36 items, JOABPEQ with 25 items, which will undoubtedly add to the burdens on clinicians during research work. Based on the above, this current study was carried out to compare the validity, reliability, and responsiveness of the JOABPEQ, NPRS, RMDQ, ODI, and SF-36 in LDH patients receiving conservative treatment to select the better option to assess the severity of disease.

## Materials and methods

### Patients and setting

LDH patients were consecutively recruited from the Longhua Hospital affiliated to Shanghai University of Traditional Chinese Medicine, and Shanghai Guanghua Hospital of Integrated Traditional Chinese and Western Medicine. To be eligible to participate in the study, participants were required to be: (1) aged 18–70 years, (2) Native Chinese speaking, (3) radiculopathy related to corresponding lumbar herniated disc with or without LBP for 1 week, radiculopathy including radicular pain, sensory abnormalities with numbness of the lower limb as the main symptom, and weakness in the distribution of one or more lumbosacral nerve roots, focal paresis, restricted trunk flexion, and increases in leg pain with straining, coughing, and sneezing are also indicative, (4) magnetic resonance imaging with single or multiple lumbar disc herniation within half a year, and (5) signed the written informed consent. Exclusion criteria included: (1) LBP with other back pathologies, such as spondylolisthesis, ankylosing spondylitis, spinal fracture, rheumatoid arthritis, secondary to tumor or other disease, (2) pregnant women, (3) patients with mental disorders, cancer and other malignant disease.

### Ethical considerations

The full study protocol was approved by the Longhua Hospital Research Ethics Committee (No. 2016LCSY030). All patients participating in the study provided informed consent.

### Questionnaires

A questionnaire booklet was constructed for the current study, including the Chinese JOABPEQ [7], NPRS, Chinese 24-item RMDQ [12], Chinese ODI [13], and Chinese SF-36 [14].

#### JOABPEQ

The JOABPEQ is developed from the original Japanese Orthopedic Association (JOA) scale for assessing LBP, which is disease specific and allows for judging patient outcome and self-administration. It is made up of 25 LBP-related items classified into five multi-item sub-scales, namely, Q1 Low back pain, Q2 Lumbar function, Q3 Walking ability, Q4 Social life function, and Q5 Mental health. The score of each factor ranges from 0 to 100 points, and a lower score is associated with worse dysfunction [15]. The five subscale scores should be used independently; adding all or some of the five subscale scores does not make sense, and summing the subscale scores to provide a total score is not necessary. The simplified Chinese version of the JOABPEQ is a reliable and valid instrument to measure functional status in patients with LBP from previous study [7].

#### NPRS

The NPRS is frequently employed to measure pain intensity, in which patients are asked to select a number (from 0 to 10) to represent their pain severity [16].

#### RMDQ

The RMDQ is a health status measure, which is designed to be completed by patients to assess their physical disability of LBP. It consists of 24 items addressing daily life and physical activity, such as personal care, sleeping, work and walking [17]. One point is assigned to each of these items, resulting in the total scores of 0 (no disability) to 24 (maximum disability) points [12].

#### ODI

The ODI is commonly used in clinical trials to measure the functional status of patients with spinal disorders [17]. It is comprised of 10 dimensions, with 6 levels being set in each dimension. Specifically, a score of 0 represents the lowest disability level, while 5 indicates the highest disability level. Moreover, the total score is converted into percentage, with a consequent maximum of 100%. Notably, version 2.1 adopted in the current study has been translated and cross-culturally adapted for Chinese patients [18].

#### SF-36

The SF-36 is composed of 8 multi-item scales, which can assess the physical function, role limitations due to physical health problems, bodily pain, general health, vitality, social functioning, role limitations due to emotional problems and emotional well-being of patients [14]. Specifically, these eight scales have been aggregated into two summary measures, which are the Physical Component Summary (PCS) score and Mental Component Summary (MCS) score [19].

### Follow-up

The patients were asked to return to the hospitals to complete the questionnaire booklet again 7–14 days after the first interview. Subsequently, all LBP scales were assessed again. The global patient evaluation (GPE) was evaluated using a 7-point Likert scale that was also completed in the second interview [20]. Besides, the response options were designed as completely recovered, much improved, slightly improved, unchanged, slightly worsened, much worsened, and worse than ever. Such scale aimed to obtain the patient ratings of improvement/deterioration as well as the importance of changes.

### Data analysis

Participants who had completed the questionnaires at baseline and 7 days later were included in the subsequent analyses. Continuous variables were summarized as the mean ± standard deviation unless otherwise noted. Data were tabulated using Microsoft EXCEL. Statistical analyses were carried out using SPSS (Version 21.0, SPSS, Gorinchem, The Netherlands). Meanwhile, the Bland-Altman method was implemented using the MedCalc statistical (Version 19.1.7, Amazon, UK).

### Internal consistency

The internal consistency of each domain was evaluated by the Cronbach’s α. In general, a Cronbach’s α of > 0.7 was acceptable [21]. All the completed baseline data were included in the analysis.

#### Test-retest reliability

The questionnaires accomplished 7 days later was tested by the intraclass correlation coefficient (ICC) (two-way random effects model, absolute agreement). Generally, an ICC of > 0.7 is recommended as a minimum standard for reliability [22]. Only patients that were rated “no change” in their global evaluation were included, since we did not propose to prevent the treatment for patients.

#### Construct validity

The relationships of the JOABPEQ, NPRS, RMDQ, ODI, and SF-36 were evaluated by means of Pearson correlation coefficients (*r*). According to Cohen’s criteria, *r* = 0.2 can be considered a small correlation, *r* = 0.5 is a medium correlation, and *r* = 0.8 is a large correlation [23].

#### Measurement error

Standard error of measurement (SEM), smallest detectable change (SDC) and Limits of Agreement (LOA) according to Bland-Altman method were used to calculate measurement error. The SEM can indicate the precision of outcome measure, which can be estimated by taking the square root of the within-subject variance of patients categorized as “unchanged” on the GPE. The SDC is calculated in accordance with 1.96*√2*SEM, which can be 95% confident that the observed change is a real change that is not caused by measurement error. The observed change represents the result of 2 measurements at baseline and follow-up, which therefore occurs twice, hence √2 [24]. The LoA was performed using Bland-Altman method, where the difference between baseline and final scores (in Y-axis) were plotted against the mean of each score at baseline and final measurement (in X-axis) [25].

#### Minimum important change (MIC)

The MIC is defined as the minimal threshold of perceptible symptom improvement that is considered as meaningful by the patients [26]. Subsequently, patients were divided into two groups based on the GPE of 7-point Likert scale, namely, the slightly improved or unchanged groups. Thus, the mean change score between two groups for the smallest meaningful change was taken as the MIC [27].

#### Responsiveness

The responsiveness has been defined as the ability of a questionnaire to detect the clinically important changes over time, even though these changes are small [28]. The responsiveness of the JOABPEQ was assessed by receiver operating characteristic (ROC) curve. In terms of the ROC curve, patients were dichotomized into four groups based on the GPE of 7-point Likert scale as completely recovered, much improved, slightly improved, or unchanged. The sensitivity values and false-positive rates (1-specificity) were plotted on the Y- and the X-axis of the curve, respectively. The area under the curve (AUC) represented the probability that a measure could correctly classify patients as clinically important improved or unchanged. An AUC of 0.7–0.8 was considered as acceptable and that of 0.8–0.9 as excellent [29].

## Results

### Patient characteristics

A total of 353 LDH patients were enrolled during a 12-month period. The mean age of patients was 50.53 ± 13.48 years and over 55% were female. The duration of the disease was 261.28 ± 327.53 weeks, 319 (90.37%) out of those 353 patients reported low back pain, 322 (91.22%) patients reported leg pain, 203 (57.51%) patients reported numbness of lower limb, 64 (18.13%) patients reported weakness of lower limb. Over half of the patients with L4/L5 level herniation (236/66.86%), and L5/S1 level herniation (195/55.24%). The baseline patient characteristics were shown in Table 1.

**Table 1:** Characteristics of patients with LDH

Characteristic	Baseline Group (***n*** = 353)	Small improve Group (***n*** = 139)	No change Group (***n*** = 65)
Age, years	50.53 ± 13.48	51.64 ± 13.29	50.13 ± 14.78
Gender, male/female	156/197	62/77	33/32
Disease duration, weeks	261.28 ± 327.53	265.19 ± 327.92	370.07 ± 408.96
Occupation, active/retired	94/259	41/98	15/49
Low back pain, *n* (%)	319 (90.37%)	125 (89.93%)	60 (92.31%)
Leg pain, *n* (%)	322 (91.22%)	126 (90.65%)	57 (87.69%)
Numbness of lower limb, *n* (%)	203 (57.51%)	75 (53.96%)	39 (60.00%)
Weakness of lower limb, *n* (%)	64 (18.13%)	21 (15.11%)	19 (29.23%)
Lesion location, *n* (%)			
L1/L2	8 (2.27%)	3 (2.16%)	0 (00.00%)
L2/L3	18 (5.10%)	7 (5.04%)	2 (3.08%)
L3/L4	57 (16.15%)	25 (17.99%)	16 (24.62%)
L4/L5	236 (66.86%)	97 (69.78%)	49 (75.38%)
L5/S1	195 (55.24%)	83 (59.71%)	44 (67.69%)
JOABPEQ			
Q1 Low back pain	31.10 ± 26.67	30.24 ± 26.16	38.55 ± 30.21
Q2 Lumbar function	44.59 ± 27.52	43.30 ± 27.07	53.65 ± 28.52
Q3 Walking ability	56.62 ± 30.46	54.92 ± 30.49	65.40 ± 29.98
Q4 Social life function	35.88 ± 21.40	36.15 ± 21.03	41.01 ± 21.89
Q5 Mental health	42.56 ± 18.88	41.52 ± 18.89	43.22 ± 17.30
NPRS	5.68 ± 2.19	6.07 ± 1.75	4.37 ± 2.06
ODI	49.57 ± 22.47	51.44 ± 21.84	45.39 ± 24.01
RMDQ	13.31 ± 6.45	13.58 ± 6.45	10.89 ± 7.07
SF-36 PCS	40.56 ± 20.47	40.08 ± 20.10	45.94 ± 21.52
SF-36 MCS	50.84 ± 21.69	50.07 ± 21.59	51.90 ± 19.87

*LDH* Lumbar disc herniation, *JOABPEQ* Japanese Orthopaedic Association Back Pain Evaluation Questionnaire, *NPRS* Numerical Pain Rating Scale, *ODI* Oswestry Disability Index, *RMDQ* Roland-Morris Disability Questionnaire, *SF-36* Short Form Health Survey, *PCS* Physical Component Summary score, *MCS* Mental Component Summary score

**Fig. 1:**
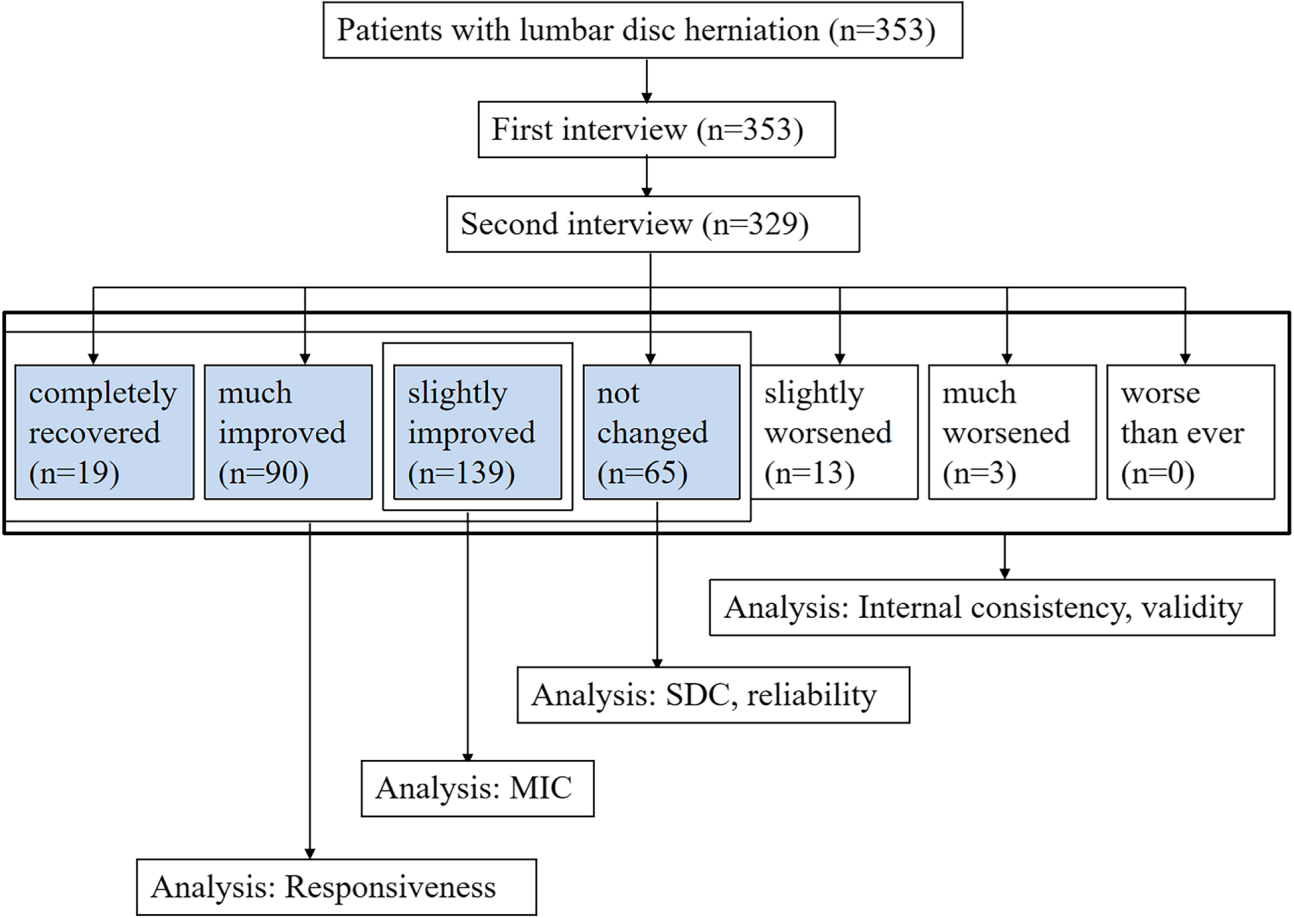
Study flow diagram; SDC, smallest detectable change; MIC, minimum important change

Finally, a total of 329 patients had completed the questionnaires twice at an interval of 8.87 ± 2.70 days, resulting in the response rate of 93.2%. Among them, 19 patients were rated as “completely recovered”, 90 as “much improved”, 139 as “slightly improved”, 65 as “unchanged”, 13 as “slightly worsened”, and 3 as “much worsened”. Meanwhile, no patient was rated as “worse than ever”. The demographic characteristics of patients were presented in Table 1, and the study flow diagram in Fig. 1.

### Data analysis

#### Internal consistency

353 LDH patients were enrolled in the internal consistency, four subscales of the Chinese JOABPEQ over 0.70 (Q1 Low back pain Cronbach’s α = 0.494, Q2 Lumbar function Cronbach’s α = 0.768, Q3 Walking ability Cronbach’s α = 0.741, Q4 Social life function Cronbach’s α = 0.701, Q5 Mental health Cronbach’s α = 0.879), indicating acceptable internal consistency, as well as other scales (ODI Cronbach’s α = 0.828, RMDQ Cronbach’s α = 0.807, SF-36 PCS Cronbach’s α = 0.774, SF-36 MCS Cronbach’s α = 0.802) (Table 2).

**Table 2:** The Crobach’s α, and ICC for the low back pain scales

	Number of item	Crobach’s α	ICC
JOABPEQ			
Q1 Low back pain	4	0.494	0.751
Q2 Lumbar function	6	0.768	0.809
Q3 Walking ability	5	0.741	0.812
Q4 Social life function	4	0.701	0.832
Q5 Mental health	7	0.879	0.866
NPRS	1	/	0.991
ODI	10	0.828	0.871
RMDQ	24	0.807	0.855
SF-36 PCS	21	0.774	0.896
SF-36 MCS	14	0.802	0.843

*ICC* intraclass correlation coefficient, *JOABPEQ* Japanese Orthopaedic Association Back Pain Evaluation Questionnaire, *NPRS* Numerical Pain Rating Scale, *ODI* Oswestry Disability Index, *RMDQ* Roland-Morris Disability Questionnaire, *SF-36* Short Form Health Survey, *PCS* Physical Component Summary score, *MCS* Mental Component Summary score

#### Test-retest reliability

65 patients showed no change were included in the test-retest analysis. The ICCs for the test-retest reliability were over 0.75 (Q1 Low back pain ICC = 0.751, Q2 Lumbar function ICC = 0.809, Q3 Walking ability ICC = 0.812, Q4 Social life function ICC = 0.832, Q5 Mental health ICC = 0.866), indicating good test-retest reliability. The other scale had similar good test-retest reliability (NPRS ICC = 0.991, ODI ICC = 0.871, RMDQ ICC = 0.855, SF-36 PCS ICC = 0.896, SF-36 MCS ICC = 0.843) (Table 2).

#### Construct validity

There were small correlations between the NPRS, and JOABPEQ (*r* = − 0.388 to − 0.457), similar with NPRS and ODI (*r* = − 0.449), RMDQ (*r* = − 0.485), and SF-36 MCS (*r* = − 0.413). Medium correlation correlations could be seen between JOABPEQ and ODI, as well as RMDQ (ODI *r* = − 0.537 to − 0.725; RMDQ *r* = − 0.597 to − 0.752), especially Q3 Walking ability and Q4 Social life function. Similar, medium correlations could also be seen between JOABPEQ and SF-36 PCS (*r* = 0.604 to 0.730) as well as SF-36 MCS (*r* = 0.517 to 0.736), especially Q5 Mental health. The Pearson correlation coefficient (*r*) of each scale were in Table 3.

**Table 3:** Pearson correlation coefficient (*r*) between each low back pain scales

Scales	Q1 Low back pain	Q2 Lumbar function	Q3 Walking ability	Q4 Social life function	Q5 Mental health	NPRS	ODI	RMDQ	SF-36 PCS	SF-36 MCS
JOABPEQ										
Q1 Low back pain	1	**0.556**^**a**^	**0.523**^**a**^	**0.580**^**a**^	0.496^a^	−0.415^a^	**−0.537**^**a**^	**− 0.627**^**a**^	**0.659**^**a**^	**0.517**^**a**^
Q2 Lumbar function	**0.556**^**a**^	1	**0.569**^**a**^	**0.679**^**a**^	**0.556**^**a**^	− 0.429^a^	**− 0.634**^**a**^	**− 0.751**^**a**^	**0.701**^**a**^	**0.553**^**a**^
Q3 Walking ability	**0.523**^**a**^	0.569^a^	1	**0.736**^**a**^	**0.535**^**a**^	−0.426^a^	**− 0.701**^**a**^	**− 0.725**^**a**^	**0.604**^**a**^	**0.536**^**a**^
Q4 Social life function	**0.580**^**a**^	**0.679**^**a**^	**0.736**^**a**^	1	**0.630**^**a**^	−0.457^a^	**− 0.715**^**a**^	**− 0.724**^**a**^	**0.730**^**a**^	**0.636**^**a**^
Q5 Mental health	0.496^a^	**0.556**^**a**^	**0.535**^**a**^	**0.630**^**a**^	1	−0.388^a^	**− 0.587**^**a**^	**− 0.597**^**a**^	**0.724**^**a**^	**0.736**^**a**^
NPRS	−0.415^a^	−0.429^a^	− 0.426^a^	−0.457^a^	− 0.388^a^	1	0.449^a^	0.485^a^	**−0.519**^**a**^	−0.413^a^
ODI	**−0.537**^**a**^	**−0.634**^**a**^	**− 0.725**^**a**^	**−0.715**^**a**^	**− 0.587**^**a**^	0.449^a^	1	**0.736**^**a**^	**−0.682**^**a**^	**−0.601**^**a**^
RMDQ	**−0.627**^**a**^	**−0.752**^**a**^	**− 0.701**^**a**^	**−0.724**^**a**^	**− 0.597**^**a**^	0.485^a^	**0.736**^**a**^	1	**−0.764**^**a**^	**−0.614**^**a**^
SF-36 PCS	**0.659**^**a**^	**0.701**^**a**^	**0.604**^**a**^	**0.730**^**a**^	**0.724**^**a**^	−0.519^a^	**−0.682**^**a**^	**− 0.764**^**a**^	1	**0.779**^**a**^
SF-36 MCS	**0.517**^**a**^	**0.553**^**a**^	**0.536**^**a**^	**0.636**^**a**^	**0.736**^**a**^	−0.413^a^	**−0.601**^**a**^	**− 0.614**^**a**^	**0.770**^**a**^	1

*JOABPEQ* Japanese Orthopaedic Association Back Pain Evaluation Questionnaire, *NPRS* Numerical Pain Rating Scale, *ODI* Oswestry Disability Index, *RMDQ* Roland-Morris Disability Questionnaire, *SF-36* Short Form Health Survey, *PCS* Physical Component Summary score, *MCS* Mental Component Summary score

According to Cohen’s criteria, *r* = 0.2 can be considered a small correlation, *r* = 0.5 is a medium correlation, and *r* = 0.8 is a large correlation

Notes

^a^Correlation is significant at the 0.01 level (2-tailed)

#### Measurement error

Sixty-five patients rated as “unchanged” were enrolled into measurement error analysis. The results suggested that the SDCs of Q1-Q5 Mental Health were ranged from 4.29 to 8.14 from SEM, then the SDCs of NPRS, ODI, RMDQ, SF-36 PCS and SF-36 MCS were 0.10, 3.43, 0.77, 3.41, and 3.93, respectively. The SDCs of the LBP scales were presented in Table 3.

**Fig. 2:**
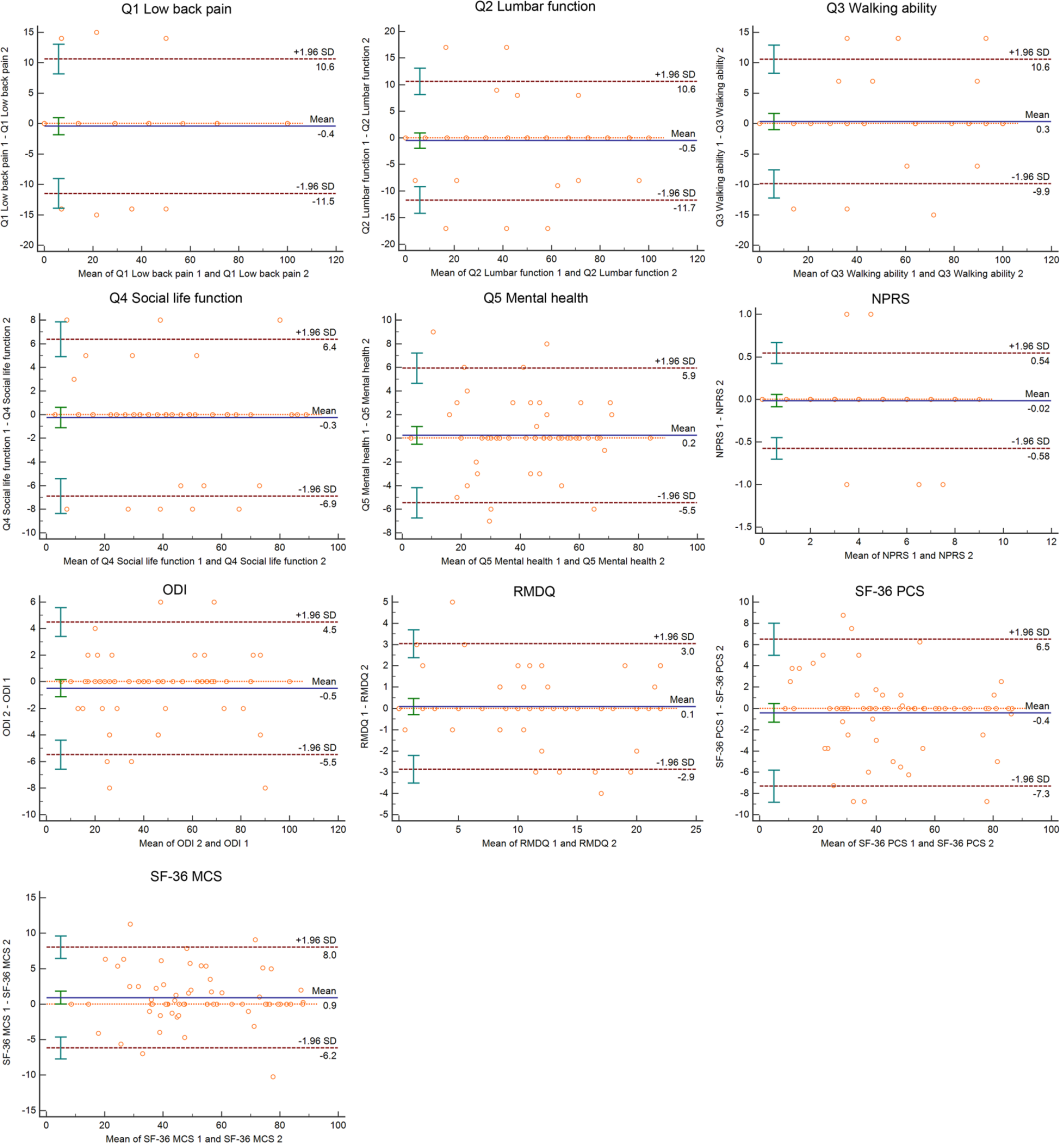
Bland-Altman plots of the low back pain scales; JOABPEQ, Japanese Orthopaedic Association Back Pain Evaluation Questionnaire; NPRS, Numerical Pain Rating Scale; ODI, Oswestry Disability Index; RMDQ, Roland-Morris Disability Questionnaire; SF-36, Short Form Health Survey; PCS, Physical Component Summary score; MCS, Mental Component Summary score

All those 65 patients were enrolled into the Bland-Altman plot, the LoAs of each LBP scales were in the Fig. 2. The LoAs of JOABPEQ ranged from 5.70 to 11.05 (Q1 Low back pain 11.05, Q2 Lumbar function 11.15, Q3 Walking ability 10.25, Q4 Social life function 6.65; Q5 Mental health 5.7. the SDCs of NPRS, ODI, RMDQ, SF-36 PCS and SF-36 MCS were 0.56, 5.0, 2.95, 6.9, and 7.1, respectively.

#### MICs

Sixty-five patients rated as “unchanged” and 139 as “slightly improved” were enrolled in MCID analysis. The results suggested that the MICs of JOABPEQ Q1–5 were 11.37 [95%CI 8.76, 13.97], 11.14 [8.69, 13.60], 11.18 [8.64, 13.72], 6.88 [5.08, 8.68], and 6.17 [4.75, 7.59], respectively.

Meanwhile, the MICs of RMDQ, ODI, NPRS, SF-36 PCS and SF-36 MCS were also calculated. The results revealed that the MICs of NPRS, ODI, RMDQ, SF-36 PCS and SF-36 MCS were 1.71 [1.52, 1.91], 5.88 [4.44, 7.31], 1.74 [1.11, 2.37], 4.57 [3.33, 5.82], and 3.59 [2.58, 4.62], respectively. The MIC and MIC% of the LBP scales were shown in Table 4.

**Table 4:** The SEM, SDC, MIC, and MIC% for the low back pain scales

Scales	Total score	SEM	SDC	MIC	MIC%
JOABPEQ					
Q1 Low back pain	100	2.94	8.14	11.37 [8.76, 13.97]	37.60%
Q2 Lumbar function	100	2.38	6.59	11.14 [8.69, 13.60]	25.73%
Q3 Walking ability	100	2.40	6.62	11.18 [8.64, 13.72]	20.36%
Q4 Social life function	100	1.59	4.40	6.88 [5.08, 8.68]	19.03%
Q5 Mental health	100	1.55	4.29	6.17 [4.75, 7.59]	14.86%
NPRS	10	0.04	0.10	1.71 [1.52, 1.91]	28.17%
ODI	100	1.25	3.43	5.88 [4.44, 7.31]	11.43%
RMDQ	24	0.28	0.77	1.74 [1.11, 2.37]	33.65%
SF-36 PCS	100	1.24	3.41	4.57 [3.33, 5.82]	11.40%
SF-36 MCS	100	1.42	3.93	3.59 [2.58, 4.62]	7.17%

*SEM* standard error of measurement, *SDC* smallest detectable change, *MIC* minimum important change, *JOABPEQ* Japanese Orthopaedic Association Back Pain Evaluation Questionnaire, *NPRS* Numerical Pain Rating Scale, *ODI* Oswestry Disability Index, *RMDQ* Roland-Morris Disability Questionnaire, *SF-36* Short Form Health Survey, *PCS* Physical Component Summary score, *MCS* Mental Component Summary score

#### Responsiveness

**Fig. 3:**
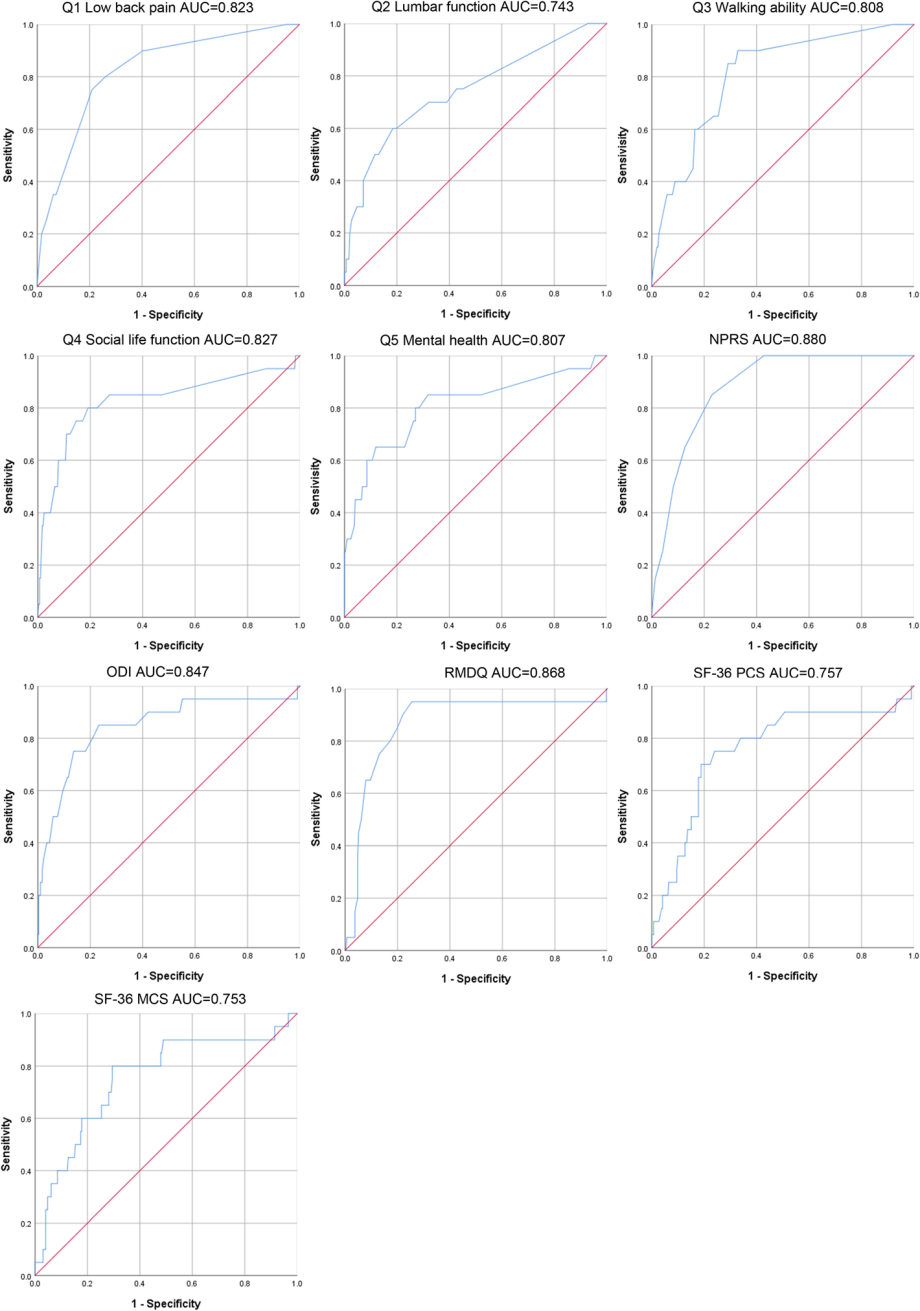
ROC curves of the low back pain scales; ROC, receiver operating characteristic; AUC, area under the curve; JOABPEQ, Japanese Orthopaedic Association Back Pain Evaluation Questionnaire; NPRS, Numerical Pain Rating Scale; ODI, Oswestry Disability Index; RMDQ, Roland-Morris Disability Questionnaire; SF-36, Short Form Health Survey; PCS, Physical Component Summary score; MCS, Mental Component Summary score

The AUCs for the responsiveness of JOABPEQ scales were presented in Table 5, and Fig. 3. As could be observed, the AUCs for JOABPEQ were ranged from 0.743 to 0.827. The results of AUC indicated that the JOABPEQ Q1 had excellent responsiveness to assess pain intensity, and JOABPEQ Q2–4 had acceptable to excellent responsiveness to assess functional status. Meanwhile, the AUCs for the responsiveness of ODI and RMDQ were over 0.80, which demonstrated excellent responsiveness to assess functional status. In addition, the AUC of NPRS was 0.880, representing excellent responsiveness to assess pain intensity; whereas those of SF-36 PCS and SF-36 MCS were 0.757 and 0.753, respectively, suggesting acceptable ability to discriminate the patients who improved and who did not related to assess quality of life.

**Table 5:** The result of the ROC analysis for the low back pain scales

Scales	AUC	Sensitivity	Specificity
JOABPEQ			
Q1 Low back pain	0.823 [0.731, 0.915]	0.750	0.791
Q2 Lumbar function	0.743 [0.619, 0.868]	0.600	0.815
Q3 Walking ability	0.808 [0.719, 0.898]	0.850	0.709
Q4 Social life function	0.827 [0.707, 0.946]	0.600	0.920
Q5 Mental health	0.807 [0.688, 0.927]	0.850	0.682
NPRS	0.880 [0.827, 0.934]	0.850	0.771
ODI	0.847 [0.741, 0.952]	0.850	0.767
RMDQ	0.868 [0.774, 0.962]	0.950	0.746
SF-36 PCS	0.757 [0.635, 0.878]	0.700	0.812
SF-36 MCS	0.753 [0.632, 0.874]	0.800	0.705

*ROC* receiver operating characteristic, *AUC* area under the curve, *JOABPEQ* Japanese Orthopaedic Association Back Pain Evaluation Questionnaire, *NPRS* Numerical Pain Rating Scale, *ODI* Oswestry Disability Index, *RMDQ* Roland-Morris Disability Questionnaire, *SF-36* Short Form Health Survey, *PCS* Physical Component Summary score, *MCS* Mental Component Summary score

## Discussion

### Study summary of this study

To the best of our knowledge, the current study is the first to test the validity, reliability, and responsiveness of the JOABPEQ, NPRS, RMDQ, ODI, and SF-36 for LDH patients receiving conservative treatment. The selection for the questionnaires in this study was based on the characteristic of LBP. The main complain of LDH are LBP, disability, and impact to the life quality. The NPRS we chose is focused on pain intensity, ODI and RMDQ are disability, and the SF-36 is the most common scale to assess quality of life. The validity, reliability, and good sensitivity of NPRS has been identified in plenty of clinical trials [16, 30, 31]. To some extent, the NPRS is superior then the other scales, such as the visual analogue scale, and verbal rating scale [31]. Then ODI, and RMDQ has been verified to be a reliable and valid LBP measurement for patients [32, 33].

It was shown that most of the scales had acceptable internal consistency, and reliability, except Q1 Low back pain in JOABPEQ. For pain intensity, small correlations between the NPRS, and other scales. For function, medium correlation could be seen between JOABPEQ and ODI, as well as RMDQ, similar, medium correlations were between JOABPEQ and SF-36 for quality of life. As the AUCs of all the scales were over 0.70, hence their responsiveness was all acceptable. In the other words, it means that for pain intensity, the NPRS could not been replaced by Q1 Low back pain, then Q2 Lumbar function, Q3 Walking ability, Q4 Social life function had the same similar performance compared with ODI, and RMDQ, then the Q5 could replace SF-36, it has higher responsiveness then SF-36, with acceptable correlation (SF-36 PCS *r* = 0.724, SF-36 MCS *r* = 0.736).

Based on the validity, reliability, and responsiveness of the LBP scales, if the studies designed to focus on pain intensity and function, the NPRS, and ODI or RMDQ are recommended, then the ODI would be more applicable to cross section survey due to a slim advantage on validity, reliability, on the contrary, RMDQ more suitable to intervention trials due to higher responsiveness in LDH patients. While if the quality of life also is needed to observe, the NPRS, and JOABPEQ would be more appropriate, as the SF-36 with 36 item results to heavier workload of researchers, and patients, and harder calculation, meanwhile our results also suggest that the SF-36 displays poorer responsiveness in LDH patients compared with other scales, which is consistent with findings from other previous studies [34, 35]. More measures are also a participant burden more than clinician or researcher burden, using more scales could make the trials more expensive for the researchers, thus this recommended could give some advice on studies related to LDH to save resources and lighten the burden of researchers and participants.

### Validity of the LBP scales

There were only small correlations between the NPRS, and JOABPEQ related to pain intensity, consistent with our previous research in LBP patients [7]. The NPRS is a single scale that only assesses pain intensity, then Q1 Low back pain is focused on the impact of pain to daily life, such as sleeping, and rest. Although both of them were designed to assess pain intensity, but with different fields. The ODI and the RMDQ are used to measure the degree of pain induced dysfunction, then Q2 Lumbar function, Q3 Walking ability, and Q4 Social life function measure the level of lumbar function, walking ability, and social life, respectively; these are important factors of pain induced dysfunction. The result was in expect that JOABPEQ has medium correlation to ODI, and RMDQ for dysfunction. The present correlations between the JOABPEQ and SF-36 are similar to those found for the Thai JOABPEQ, and our previous research in LBP patients, especially for Q5 Mental health, suggesting that this item can objectively assess the life quality of patients with LBP [7, 9].

### The MICs and responsiveness of the JOABPEQ

Currently, the JOABPEQ has also been translated into Arabic [36], Thai [9], Turkish [8], and Persian [37]. Nonetheless, only the original version, has explored the MIC or responsiveness of the JOABPEQ. The MIC of the original JOABPEQ ranged from 27.9 points (Q3 Walking ability) to 14.8 points (Q5 Mental health) in patients with LDH after discectomy ([38]Azimi P 2018), then it was ranged from 28.5 points (Q1 Low back pain) to 14.5 points (Q5 Mental health) in patients with decompression surgery for lumbar spinal stenosis ([39]Ogura Y 2019). In our study, the MICs of the simplified Chinese JOABPEQ ranged from 11.37 to 6.17 points for LDH, are smaller than the recommended by the original JOABPEQ. The trend was the same, the MICs of the Q1 to Q3 were larger than Q4 and Q5 Mental health, then the responsiveness were similar in both simplified Chinese and original version ([38] Azimi P 2018, [39] Ogura Y 2019, [40] Fujimori T). It was suspected that the patients in surgery group might had greater expectations for treatment rather than conservative treatment. In other words, the patient with higher improvement would rate slightly improved or much improved with surgery than conservative treatment. The same situation occurred in other disease, such as the American Shoulder and Elbow Surgeons (ASES) score and Simple Shoulder Test (SST) score rotator cuff disease with arthroscopic rotator cuff repair (ASES 20.9; SST 2.4) or arthroplasty (ASES 27.1; SST 4.3) or conservative treatment (ASES 12.01; SST 2.05) ([41] Tashjian RZ 2020, [42] Tashjian RZ 2016, [43] Tashjian RZ 2010). Then it was found that the patients accepted discectomy was more serious than the patients in our study, that might give the patients more space to improve. Thus the test of MIC and responsiveness is expected to be conducted in other different populations with receiving conservative treatment.

### Superiorities and limitations

The use of GPE is controversial for most studies of this type, and the validity of a single-item design compared with a multi-items scale is also doubtful [44]. However, such limitations are inevitable. Moreover, the GPE is also associated with another disadvantage, which is that it may be difficult for patients to recall their initial health status and to compare it with their current status to assess any changes. Therefore, this may introduce bias [45]. In the current study, patients are asked to return to hospitals to complete the questionnaire booklet again 7–14 days after the first interview. This seems not to be a long time, which may be easier for patients to recall their initial health status. Secondly, treatments such as acupuncture and manual therapies are allowed for LBP patients, which have favorable short-term effects on them.

Furthermore, a small proportion of participants are retested within a short interval. Therefore, they may have been biased to give the same answer if they have remembered some of the questions asked at the first time, even though there are 96 items of these questionnaires.

## Conclusion

Based on the validity, reliability, and responsiveness of the LBP scales, if the studies focus on pain intensity and function, the NPRS, and ODI for cross section survey or RMDQ intervention trials are recommended in LDH patients. While if the quality of life also is needed to observe, the NPRS, and JOABPEQ would be more appropriate rather than SF-36. This recommended could give some advice on studies related to LDH to save resources and lighten the burden of researchers, as well as participants.

## Data Availability

The datasets used and/or analysed during the current study are available from the corresponding author on reasonable request.
